# Vitamin C as a Potential Interplaying Factor between Obesity and COVID-19 Outcome

**DOI:** 10.3390/healthcare11010093

**Published:** 2022-12-28

**Authors:** Wael Hafez, Husam Saleh, Mahmoud Abdelshakor, Shougyat Ahmed, Sana Osman, Muneir Gador

**Affiliations:** 1 NMC Royal Hospital, 16th Street, Khalifa City, Abu Dhabi P.O. Box 764659, United Arab Emirates; 2 The Medical Research Division, Department of Internal Medicine, The National Research Center, El Buhouth Street, Ad Doqi, Cairo 12622, Egypt

**Keywords:** COVID-19, vitamin C, body mass index, obesity, severity, viral clearance

## Abstract

(1) Background: Obesity is a risk factor for severe COVID-19 outcomes. Obesity can interfere with the action of vitamin C. The study aimed to investigate the association between BMI and severe outcomes of COVID-19 while considering vitamin C levels; (2) Methods: This was a retrospective study of 63 COVID-19 patients treated at the NMC Royal Hospital, United Arab Emirates; (3) Results: There was no significant difference in vitamin C levels among patients in all BMI categories (*p* > 0.05). The risk of severe COVID-19 significantly increased by 5.4 times among class 1 obese patients compared with normal BMI (OR = 5.40, 95%CI: (1.54–21.05), *p* = 0.010). Vitamin C did not affect the risk of COVID-19 severity or mortality across BMI categories (*p* = 0.177, *p* > 0.05, respectively). The time till viral clearance was significantly different among patients in different BMI categories when vitamin C levels were considered (*p* = 0.02). Although there was no significant difference in vitamin C levels across BMI categories, there was a significant interaction between vitamin C levels and viral clearance rate in obese patients; (4) Conclusions: Given the safety of vitamin C supplements and our findings, proper vitamin C uptake and supplementation for patients of various BMI levels are encouraged.

## 1. Introduction

Coronavirus disease 2019 (COVID-19) is an infectious disease that emerged recently as a case series of pneumonia in Wuhan, China, at the end of 2019. The causative organism was also isolated and named severe acute respiratory syndrome coronavirus-2 (SARS-CoV-2) [1,2]. Severe COVID-19 outcomes result from hyper-inflammation, macrophage activation, and cytokine storm [3].

Several risk factors for severe COVID-19 outcomes include hypertension, diabetes, cardiovascular diseases, smoking, and obesity [4]. Several studies have reported obesity as an independent risk factor for prolonged hospitalization, severity, Intensive Care Unit (ICU) admission, need for mechanical ventilation, and death among COVID-19 patients [5,6,7].

Obesity is characterized by the disruption of innate and adaptive immune responses and the presence of chronic low-grade inflammation, which results from impaired metabolism. Chronic inflammation is also associated with a decline in macrophage activity and increased production of pro-inflammatory cytokines, leading to increased susceptibility and delayed shedding during viral infection [8].

Vitamin C is an antioxidant that plays an important role in preventing several diseases by protecting against oxidative stress. The immune-regulatory roles of vitamin C include enhanced neutrophil migration and chemotaxis, proliferation, differentiation, maturation of T cells, and inhibition of pro-inflammatory cytokines production [9]. 

An association between optimal vitamin C status and improved pneumonia and ICU admission outcomes was observed [10,11]; however, the replication of these findings in the context of COVID-19 is still controversial. Still, it was advised to administer a dose of 1.5 g/kg of body weight to COVID-19 patients as prophylaxis, which is considered a safe dose without any adverse events [3].

A previous study by Wilson et al. reported body mass index (BMI) as an independent predictor for serum vitamin C concentration. They described a significantly negative association between vitamin C concentration and BMI, which may be due to oxidative stress and chronic inflammation. These findings suggest that patients with increased weight may require a higher intake of vitamin C to achieve appropriate plasma concentrations [12].

The current study aimed to investigate the association between BMI and severe outcomes of COVID-19 while considering the serum levels of vitamin C.

## 2. Materials and Methods

### 2.1. Study Design and Population

This study was a non-interventional retrospective study of COVID-19 patients’ medical records. The study was conducted in NMC Royal Hospital, Khalifa City, Abu Dhabi, United Arab Emirates (UAE) during the first pandemic wave. 

COVID-19 diagnosis was confirmed by reverse transcriptase-polymerase chain reaction real-time (RT-PCR) assay—Solgent’s 2019-nCoV RT-PCR Kit—using nasopharyngeal swabs under aseptic operation. Included patients were hospitalized 63 COVID-19 patients with different disease severity grades.

Patients Identifiers were removed during the data collection process, with complete protection of patients’ privacy. This study was accompanied based on the Declaration of Helsinki. This study was reviewed and approved by the Regional Research Ethics Committee, department of health, Abu Dhabi, UAE (ADHRTC-2021-180).

### 2.2. Inclusion and Exclusion Criteria

Adult patients with COVID-19 aged 18 or above were included, while patients who previously received vitamin C supplementation before hospital admission were excluded. 

### 2.3. Vitamin C Analysis

Measurement of serum vitamin C levels was performed at the National Reference Laboratory (UAE) under order code (001805); the test was done using liquid chromatography-tandem mass spectrometry (LC/MS-MS). The cut-off value was 0.4 mg/dl, plasma levels less than 0.4 mg/dl were defined as sufficient, and values above 0.4 mg/dl were recognized as normal. The test was developed by LabCorp Burlington, 1447 York Cort, Burlington, NC 27215-3361.

### 2.4. Data Collection

Demographic, clinical, and biochemical data were retrospectively retrieved from the hospital system INSTA, including gender, age, BMI, co-existing diseases, epidemiology, and clinical symptoms of all patients obtained from the electronic medical records. Laboratory and radiological findings, therapeutic interventions, and disease outcomes were also extracted. 

BMI is classified into underweight (BMI < 18.5), normal (BMI is 18.5 to <25), overweight (BMI is 25.0 to <30), and obese (BMI is 30.0 or higher). Obesity could also be divided into 3 classes; Class 1 (BMI of 30 to <35), Class 2 (BMI of 35 to <40), and Class 3 (BMI of 40 or higher) [13].

Baseline laboratory investigations were done at the time of and during admission, including inflammatory markers indicative of severe disease (platelet, lymphocyte count, C-reactive protein (CRP), Lactate dehydrogenase (LDH), Fibrinogen, D-dimer, complete blood count, liver function tests, kidney function tests, lymphocytic count, and serum ferritin. According to the clinical assessment, all patients had a chest x-ray and/or chest CT on presentation and during follow-up within different interval times 

Prophylactic anticoagulation was advised for all severe COVID-19 cases and was increased to therapeutic levels in cases of progressive deterioration and/or progressive increase in D-dimer levels. The time interval between the first positive and the first negative PCR test for two consecutive negatives was defined as the time till viral clearance.

### 2.5. Statistical Analysis

After data collection and verification, all data were entered for statistical analysis using R Software version 3.5.2 (2018-12-20)—“Eggshell Igloo”, and the appropriate statistical tests were carried out. Quantitative data with normal distribution were presented as mean ± standard deviation (±SD) and range; when normal distribution was violated, data were presented as median and interquartile range. Qualitative data were presented as frequency (n) and percentage (%).

Univariate analysis was performed to compare patients with different BMI classes regarding their baseline demographic, clinical, and biochemical characteristics using the chi-square test, t-test, or Wilcoxon rank-sum test as appropriate. The univariate logistic regression model was performed to examine the association between BMI classes of COVID-19 patients and vitamin C serum levels. In addition, univariate and multivariate logistic regression was used to investigate the association between BMI and COVID-19 severity and mortality when different serum levels of vitamin C were considered. Survival analysis was also done to specify the association between BMI and time till viral clearance using the Kaplan Meier curve and comparison using the Log-rank test while considering different serum levels of vitamin C. Unadjusted and adjusted hazard measurements using the COX regression model were performed when appropriate. COX regression model 1 was done while considering the normal BMI cut point as below 30. COX regression model 2 was performed while considering the normal BMI (BMI < 25), overweight (25 < BMI < 30), and obesity (BMI ≥ 30). The confidence interval was set to 95%, and the margin of error accepted was 5%. So, the p-value was considered significant as the following: *p* > 0.05: Non-significant (NS), *p* < 0.05: Significant (S), and *p* < 0.01: Highly significant (HS).

## 3. Results

### 3.1. Demographic and Clinical Characteristics of the Study Population

The current study included 63 COVID-19 patients treated at NMC Royal Hospital. The majority of the study participants (79.3%) were males, and 69.8% were Asians. About 69.8% of the study population had normal BMI, 22.2% were classified as obesity class 1, 4.8% as class 2, and 3.2% as class 3. 

**Figure 1:**
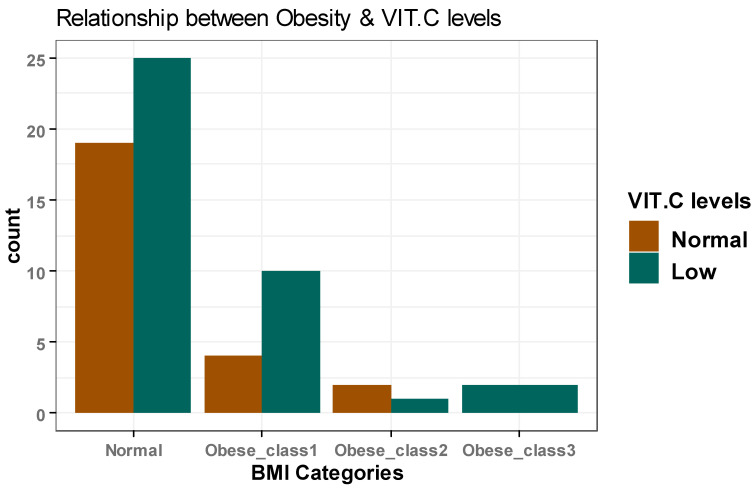
The association between BMI classes and serum vitamin C levels.

There was a statistically significant increase in CRP, LDH, AST, and Ferritin (*p* = 0.009, 0.022, 0.048, 0.008, respectively) in patients classified as obese class 1 compared with those with normal BMI. However, the patients’ remaining demographic and clinical characteristics did not significantly differ among all BMI categories (Table 1). Additionally, there was no significant difference in vitamin C serum levels among patients in all BMI categories (*p* > 0.05) (Table 2) (Figure 1).

### 3.2. The Effect of Vitamin C Levels on the Correlation between BMI and COVID-19 Severity

The risk of severe COVID-19 had been significantly increased by 5.4 times among patients classified as obesity class 1 compared with those with normal BMI (OR = 5.40, 95%CI: (1.54–21.05), *p* = 0.010). Vitamin C had no effect on the risk of COVID-19 severity among patients in all BMI categories (*p* = 0.177) (Table 3). 

**Table 3:** Univariate and multivariate logistic regression investigating the association between BMI and COVID-19 severity while considering serum levels of vitamin C (odds ratio).

		Non- Severe	Severe	Unadjusted OR (95%CI)	*p* Value
BMI Categories	Normal	33 (80.5%)	11 (50.0%)	Reference group	
	Obese_class1	5 (12.2%)	9 (40.9%)	5.40 (1.54–21.05)	0.010
	Obese_class2	2 (4.9%)	1 (4.5%)	1.50 (0.07–17.20)	0.750
	Obese_class3	1 (2.4%)	1 (4.5%)	3.00 (0.11–80.29)	0.451
VIT.C levels	Normal	21 (47.7%)	7 (30.4%)	Reference group	0.177
	Low	23 (52.3%)	16 (69.6%)	2.09 (0.74–6.36)	

**Figure 2:**
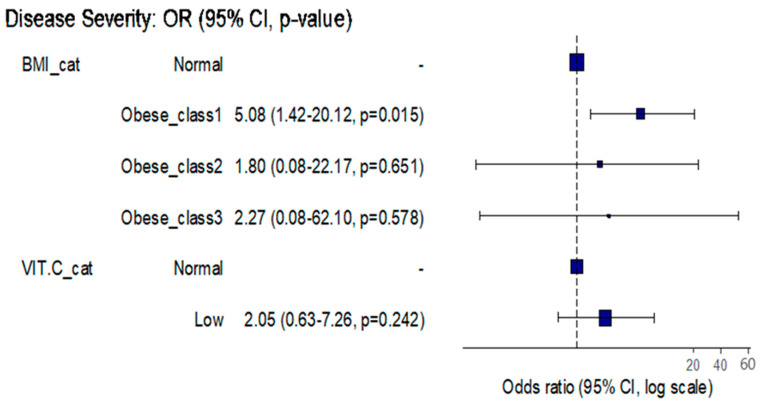
The association between BMI classes COVID-19 severity while considering different serum vitamin C levels.

So, the risk of severe COVID-19 remained significantly increased by 5.08 times among obesity class 1 patients when compared to those with normal BMI even after controlling for the effect of vitamin C serum levels (OR = 5.08, 95%CI: (1.42–20.12), *p* = 0.015) (Figure 2).

### 3.3. The Effect of BMI and Vitamin C Levels on COVID-19 Mortality

There was no statistically significant effect of patients’ BMI on COVID-19 mortality. Additionally, vitamin C serum levels did not show any significant effect on the risk of disease mortality (*p* > 0.05) (Table 4).

### 3.4. Time Till Viral Clearance among COVID-19 Patients with Different Vitamin C Levels in All BMI Categories 

According to the Kaplan Meier estimator, the median time till viral clearance in patients with normal BMI and normal vitamin C levels was (22 days, 95%CI: (15–31)). While in those with normal BMI and low vitamin C levels, the median time was (13 days, 95%CI: (9–20)).

In obesity class 1, the median time till viral clearance was 14.5 days and 27 days in patients with normal vitamin C and low vitamin C levels, respectively. In obesity class 2, the median time till viral clearance was 31.5 days and 16 days in patients with normal vitamin C and low vitamin C levels, respectively. Additionally, in obesity class 3, the median time till viral clearance was 9 days in patients with low vitamin C levels.

**Figure 3:**
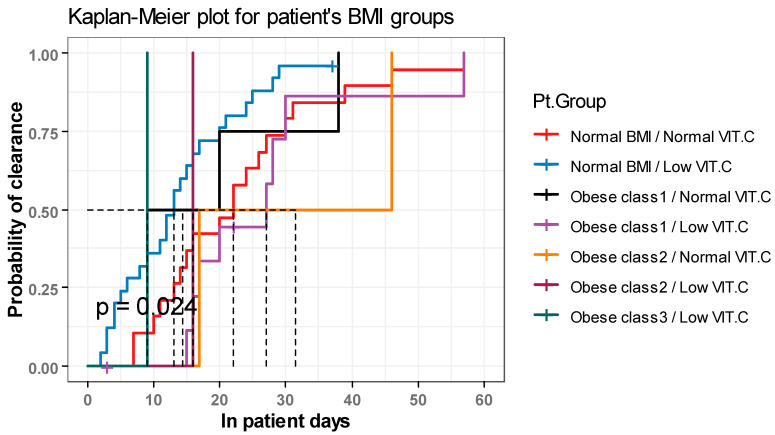
Survival analysis investigating the association between BMI classes and time till viral clearance using Kaplan Meier approach when different levels of vitamin C were considered.

The time till viral clearance was significantly different among COVID-19 patients in different BMI categories when vitamin C serum levels were considered (*p* = 0.02, log-rank test = 10.98) (Figure 3).

### 3.5. The Effect of BMI and Vitamin C Levels on the Rate of Viral Clearance

According to the adjusted Cox proportional hazard model 1, class 1 obesity patients with low vitamin C levels showed a significantly lower rate of viral clearance by about 76% decrease compared with those with normal BMI and normal vitamin C levels (RR = 0.242, 95%CI: (0.06–0.97), *p* = 0.045) (Table 5). 

These results indicate that vitamin C levels had a significant interaction effect on the viral clearance rate of higher BMI patients.

**Figure 4:**
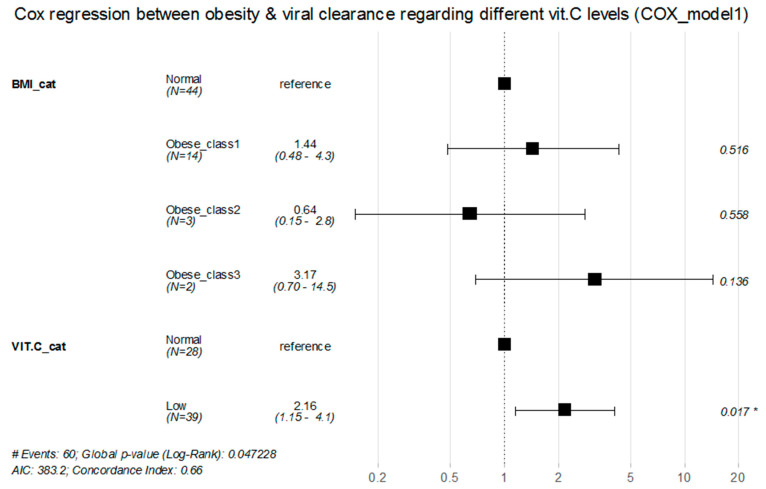
Cox proportional hazard regression for the association between obesity classes and rate of viral clearance while considering different vitamin C levels (Cox regression model 1). * Significant.

**Figure 5:**
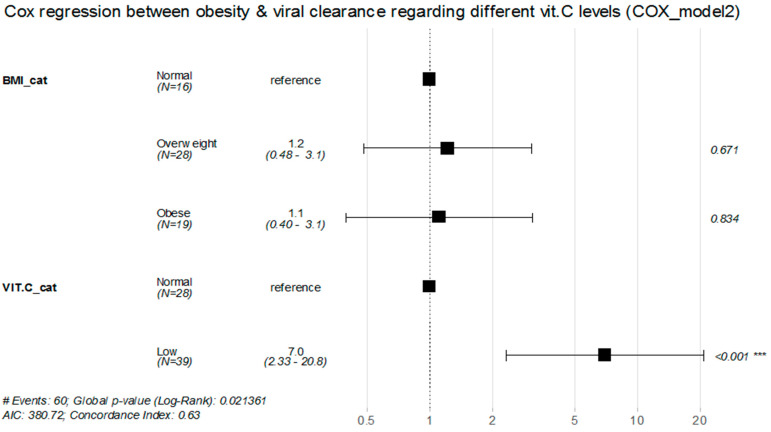
Cox proportional hazard regression for the association between obesity and rate of viral clearance while considering different vitamin C levels (Cox regression model 2). *** Vey high Significant.

However, the univariate model analysis showed no statistically significant effect for patients’ BMI alone or vitamin C serum levels alone on the rate of viral clearance (*p* values > 0.05) (Figure 4 and Figure 5).

## 4. Discussion

The current study investigated the association between obesity and COVID-19 outcomes while considering the serum levels of vitamin C. There was an increased risk of severe COVID-19 outcomes among patients with class 1 obesity. However, the risk of mortality was not significant among all BMI classes. Vitamin C levels did not affect the risk of COVID-19 severity or mortality. 

The association between obesity and COVID-19 is still not well understood. In a cohort study by Gao et al., an elevation of BMI of more than 23 Kg/m^2^ was associated with an increased risk of COVID-19 severity. The authors found no effect for sex and being Asian or Chinese on the risk of severity, but young age and black ethnicity increased the risk of COVID-19 severity. Additionally, they reported a J-shaped association between BMI and mortality due to COVID-19 risk in patients with a BMI higher than 28 kg/m^2^, which is not consistent with our findings and could be attributed to the small sample size of our cohort [14].

Another nationwide study in Korea showed a U-shaped association between BMI and COVID-19 fatality, and BMI ≥ 25.0 kg/m^2^ and < 18.5 kg/m^2^ were shown to be independent risk factors for COVID-19-associated mortality [15]. Anderson et al. observed that class 3 obesity was associated with an increased risk of intubation and mortality due to COVID-19, especially among patients younger than 65 years [16]. 

A large cohort study based on the UK biobank found a dose-dependent association between BMI and waist circumference with the risk of infection with SARS-CoV-2 and death [17,18]. However, genetically predicted BMI and not waist circumference showed a significant association with disease susceptibility based on a two-sample multivariable Mendelian randomization study [19]. 

While Wolf et al. reported no significant difference between all BMI classes regarding respiratory physiology, ICU stay duration, mechanical ventilation, and mortality due to COVID-19 [20]. Another multi-ethnicity study in UAE showed no association between obesity mortality due to COVID-19, which is consistent with our findings in the same population [21]. 

There are several molecular and cellular dysfunctions caused by obesity that could contribute to the pathogenesis of COVID-19. The overexpression of angiotensin-converting enzyme-2 receptor (ACE-2) was observed among obese patients, and recently it has been linked with higher SARS-CoV-2 infection and replication. Dysregulation of the mTOR signaling pathway also seems to increase viral replication. Other possible molecular mechanisms include endoplasmic reticulum stress and dysregulated Unfolded Protein Response (UPR) pathway, dysregulation of renin-angiotensin (RAS) pathway, metabolic reprogramming of the host cells, altered immune response, adiponectin/leptin imbalance, and endothelial dysfunction [22]. 

Previously, sufficient vitamin C levels were associated with lower incidences of chronic respiratory diseases and pneumonia [10]. Early observational studies of COVID-19 suggested vitamin C supplementation as a key modality in managing COVID-19. It was observed that patients who received vitamin C had a high recovery rate, improved oxygen status, a decline in inflammatory markers, and decreased mortality. However, similar findings were not obtained in the clinical trials [23].

The discrepancy of findings regarding the role of vitamin C supplementations in COVID-19 patients might be attributed to the difference in the dose and route of administration of it, leading to insufficient vitamin C levels among patients. As it was shown that the IV administration might quickly reach the therapeutic level with 30–70 times higher plasma concentration peak compared to oral administration [24]. 

Additionally, many studies confirmed that the high dose of intravenous vitamin C (HDIVC) infusion was more effective against severe COVID-19 in different settings [25,26,27].

There is an inverse relationship between vitamin C levels and BMI. Previous supplementation studies showed an attenuated response to vitamin C supplementation in patients with increased body weight, suggesting that obese patients may have higher requirements for vitamin C [28]. 

Several hypotheses may explain this relationship between BMI and vitamin C levels, including increased requirements with increasing BMI, metabolic dysfunction, decreased absorption, sequestration within adipose tissue, and low-grade inflammation [29]. In addition, previous studies suggested that vitamin C is capable of eliminating different manifestations of vitamin K deficiency [30], which is concentrated in adipose tissue and decreased with higher adiposity, suggesting that vitamin C might exert its positive action by relieving the complications of vitamin k deficiency in obese individuals [31]. Vitamin C deficiency among patients with high BMI was previously reported [32], and it could be partly attributed to a volumetric dilution effect [33].

Previous reports reported prolonged time till viral clearance among overweight and obese COVID-19 patients [34,35,36]. Here, we show for the first time that the interaction between high BMI and vitamin C deficiency is associated with a prolonged time till viral clearance. 

When vitamin C levels were considered, the time till viral clearance was significantly different among patients with different BMI classes. Class 1 obesity with low vitamin C levels was associated with a slower viral clearance rate. Additionally, being overweight or obese with low vitamin C levels was associated with a decline in the viral clearance rate, indicating that vitamin C levels had a significant interaction effect on the viral clearance rate of higher BMI patients.

In a matched before and after retrospective study, there was no significant difference in the time till viral clearance in the high-dose intravenous vitamin C treatment group and control group; however, BMI was not calculated in this study [37].

The limitations of our study include the limited sample size, the retrospective study design, the imbalanced distribution of the study population, the role of other micronutrients was not assessed, and the role of confounding factors such as age, sex, ethnicity, and the presence of other co-morbidities was not adjusted.

## 5. Conclusions

In summary, there was no significant difference in the serum levels of vitamin C in our COVID-19 study population. Serum vitamin C levels did not affect the association between BMI and COVID-19 severity and mortality. However, the time till viral clearance was different among all BMI classes when vitamin C levels were considered, indicating that vitamin C had a significant interplaying effect on the viral clearance rate among patients with higher BMI. Further larger controlled clinical trials are required to understand the causal association between obesity and COVID-19 and the impact of vitamin C supplementation on the outcomes of obese COVID-19 patients. Additionally, the adequate dose and proper route for vitamin C supplementation to achieve optimal serum concentration among patients with different BMI should be investigated and determined.

## Figures and Tables

**Table 1:** Univariate analysis comparing the BMI classes of COVID-19 patients regarding their baseline demographic, clinical, and biochemical characteristics.

		Normal 44 (69.8%)	Obese Class1 14 (22.2%)	Obese Class2 3 (4.8%)	Obese Class3 2 (3.2%)	*p* Value
Demographic						
Age (years)	Mean ± SD	40.3 ± 13.2	45.2 ± 12.3	43.3± 6.4	51.0 ± 17.0	0.465
Sex	Female	9 (69.2%)	3 (23.1%)	0 (0.0%)	1 (7.7%)	0.59
	Male	35 (70.0%)	11 (22.0%)	3 (6.0%)	1 (2.0%)	
Race	Asian	33 (75.0%)	8 (18.2%)	2 (4.5%)	1 (2.3%)	0.576
	Black	2 (66.7%)	1 (33.3%)	0 (0.0%)	0 (0.0%)	
	White	9 (56.2%)	5 (31.2%)	1 (6.2%)	1 (6.2%)	
Clinical						
HTN	No	40 (75.5%)	9 (17.0%)	2 (3.8%)	2 (3.8%)	0.072
	Yes	4 (40.0%)	5 (50.0%)	1 (10.0%)	0 (0.0%)	
DM	No	37 (75.5%)	9 (18.4%)	2 (4.1%)	1 (2.0%)	0.164
	Yes	7 (50.0%)	5 (35.7%)	1 (7.1%)	1 (7.1%)	
CVD	No	43 (70.5%)	13 (21.3%)	3 (4.9%)	2 (3.3%)	0.516
	Yes	1 (50.0%)	1 (50.0%)	0 (0.0%)	0 (0.0%)	
Biochemical						
VITA.C.LEVEL (mg/dl)	Mean ± SD	0.4 ± 0.4	0.3 ± 0.3	0.5 ± 0.4	0.1 ± 0.0	0.468
WBC (×10^9^/L)	Mean ± SD	6.6 ± 2.4	7.4 ± 4.6	9.6 ± 3.7	11.1 ± 5.4	0.107
Hemoglobin (g/L)	Mean ± SD	14.6 ± 4.8	13.1 ± 2.0	14.4 ± 0.6	13.2 ± 3.3	0.716
Platelets (×10^9^/L)	Mean ± SD	319.8 ± 132.6	364.3 ± 160.4	324.7 ± 183.5	485.0 ± 295.6	0.37
CRP (mg/L)	Mean ± SD	52.7 ± 85.2	154.4 ± 122.0	119.0 ± 119.8	95.5 ± 6.4	0.009
D.DIMER (µg/mL)	Mean ± SD	1.7 ± 5.3	3.7 ± 7.0	1.0 ± 1.3	1.1 ± 0.0	0.661
IL6 (pg/mL)	Mean ± SD	56.7 ± 83.5	219.5 ± 400.9	-	-	0.313
LDH (U/L)	Mean ± SD	302.1 ± 146.3	579.4 ± 515.8	408.3 ± 364.6	347.5 ± 191.6	0.022
ALT (U/L)	Mean ± SD	48.8 ± 35.5	66.9 ± 51.1	67.3 ± 52.2	25.0 ± 5.7	0.322
AST (U/L)	Mean ± SD	45.4 ± 25.4	75.8 ± 57.9	61.7 ± 43.3	34.5 ± 12.0	0.048
Creatinine (mg/dL)	Mean ± SD	2.7 ± 11.9	0.8 ± 0.3	0.9 ± 0.1	1.3 ± 0.4	0.936
Neutrophil Count (×10^9^/L)	Mean ± SD	62.2 ± 15.5	61.9 ± 17.7	58.7 ± 11.2	78.7 ± 4.9	0.515
Lymphocyte Count (×10^9^/L)	Mean ± SD	27.9 ± 13.4	28.1 ± 14.3	30.2 ± 10.9	14.1 ± 6.1	0.54
NLR	Mean ± SD	4.2 ± 5.8	3.9 ± 4.7	2.2 ± 1.1	6.2 ± 3.0	0.877
RDW.CV (%)	Mean ± SD	13.6 ± 2.6	14.4 ± 3.0	13.3 ± 1.3	13.1 ± 0.1	0.76
Fibrinogen (mg/dL)	Mean ± SD	497.7 ± 208.8	678.6 ± 199.2	592.7 ± 338.3	568.5 ± 282.1	0.06
Ferritin (ng/mL)	Mean ± SD	528.6 ± 596.8	1462.0 ± 1537.7	562.7 ± 442.2	100.5 ± 0.7	0.008
COOMB.TEST	Negative	37 (72.5%)	9 (17.6%)	3 (5.9%)	2 (3.9%)	0.320
	Positive	7 (58.3%)	5 (41.7%)	0 (0.0%)	0 (0.0%)	
ADAM.3T3 (ng/ml)	Mean ± SD	67.1 ± 22.9	54.5± 16.9	64.6 ± 30.8	79.0 ± NA	0.611
BLOOD.GROUP	A	10 (83.3%)	1 (8.3%)	1 (8.3%)	0 (0.0%)	0.413
	AB	2 (50.0%)	2 (50.0%)	0 (0.0%)	0 (0.0%)	
	B	9 (90.0%)	1 (10.0%)	0 (0.0%)	0 (0.0%)	
	O	13 (68.4%)	4 (21.1%)	0 (0.0%)	2 (10.5%)	
RH	Negative	12 (60.0%)	6 (30.0%)	2 (10.0%)	0 (0.0%)	0.283
	Positive	32 (74.4%)	8 (18.6%)	1 (2.3%)	2 (4.7%)	
VITA.D.LEVEL (ng/mL)	Mean ± SD	27.5 ± 27.1	20.1 ± 8.0	16.1 ± 0.9	41.2 ± 29.5	0.539
PT (seconds)	Mean ± SD	14.4± 1.3	14.1± 1.6	13.3 ± 1.5	-	0.429
INR	Mean ± SD	1.0 ± 0.1	1.1 ± 0.1	1.0 ± 0.1	1.0 ± 0.1	0.613
SE.LEVEL (ng/mL)	Mean ± SD	117.7 (22.4)	111.0 (2.8)	-	-	0.704
TROP.I	Mean ± SD	0.015 ± 0.037	0.0089 ± 0.0089	0.014 ± 0.01	0.006± 0.0057	0.905
PCT (ng/mL)	Mean ± SD	0.2 ± 0.5	0.2 ± 0.4	0.8± 1.1	0.0 ± NA	0.358
GLU (mmol/L)	Mean ± SD	6.0 ± 1.6	8.1 ± 4.3	7.5± 2.6	6.8 ± 3.3	0.141

CVD, Cardiovascular Diseases.

**Table 2:** Univariate logistic regression investigating the association between BMI and vitamin C serum levels (odds ratio).

		Normal VIT. C Level	Low VIT. C Level	OR (95%CI)	*p* Value
BMI Categories	Normal	19 (76.0%)	25 (65.8%)	Reference group	
	Obese_class1	4 (16.0%)	10 (26.3%)	1.90 (0.54–7.79)	0.335
	Obese_class2	2 (8.0%)	1 (2.6%)	0.38 (0.02–4.25)	0.443
	Obese_class3	0 (0.0%)	2 (5.3%)	11895034.20 (0.00–NA)	0.992

**Table 4:** Univariate and multivariate logistic regression investigating the association between BMI and COVID-19 mortality while considering serum levels of vitamin C (odds ratio).

		Improved	Died	Unadjusted OR (95%CI)	*p* Value
BMI Categories	Normal	43 (71.7%)	1 (33.3%)	Reference group	
	Obese_class1	12 (20.0%)	2 (66.7%)	7.17 (0.63–162.22)	0.120
	Obese_class2	3 (5.0%)	0 (0.0%)	0.00 (NA–Inf)	0.997
	Obese_class3	2 (3.3%)	0 (0.0%)	0.00 (NA–Inf)	0.997
VIT.C levels	Normal	28 (43.8%)	0 (0.0%)	Reference group	0.996
	Low	36 (56.2%)	3 (100.0%)	71211285.78 (0.00–NA)	

**Table 5:** Univariate and multivariate Cox proportional hazard regression for the association between obesity classes and rate of viral clearance while considering different vitamin C levels (Cox regression model 1).

		Unadjusted HR (95%CI)	*p* Value	Adjusted HR (95%CI)	*p* Value
BMI Categories	Normal	Reference group		Reference	
	Obese_class1	0.64 (0.34–1.22)	0.172	1.44 (0.48–4.28)	0.516
	Obese_class2	0.61 (0.19–2.00)	0.418	0.644 (0.148–2.8)	0.5576
	Obese_class3	4.31 (0.96–19.35)	0.056	3.17 (0.697–14.458)	0.1355
VIT.C levels	Normal	Reference group		Reference	
	Low	1.49 (0.90–2.47)	0.122	2.16 (1.147–4.067)	0.017 *
Obese_class1 and Low VIT.C		-	-	0.242 (0.06–0.97)	0.045 *
Obese_class2 and Low VIT.C		-	-	1.516 (0.125–18.35)	0.744
Obese_class3 and Low VIT.C		-	-	-	-

Similar findings were observed in Cox proportional hazard model 2, as overweight or obese COVID-19 patients with low vitamin C levels had significantly lower rates of viral clearance by about 78% and 86% decrease, respectively, when compared with those with normal BMI and normal vitamin C levels ((RR = 0.22, 95%CI: (0.057–0.84), *p* = 0.026), (RR = 0.14, 95%CI: (0.032–0.5995), *p* = 0.008), respectively) (Table 6). * significant.

**Table 6:** Univariate and multivariate Cox proportional hazard regression for the association between obesity and rate of viral clearance while considering different vitamin C levels (Cox regression model 2).

		Unadjusted HR (95%CI)	*p* Value	Adjusted HR (95%CI)	*p* Value
BMI Categories	Normal	Reference group		Reference	
	Overweight	0.95 (0.50–1.79)	0.873	1.22 (0.484–3.089)	0.67
	Obese	0.69 (0.34–1.37)	0.283	1.12 (0.396–3.15)	0.83
VIT.C levels	Normal	Reference group		Reference	
	Low	1.49 (0.90–2.47)	0.122	6.96 (2.33–20.79)	<0.001 ***
Overweight and Low VIT.C		-	-	0.22 (0.057–0.84)	0.026 *
Obese and Low VIT.C		-	-	0.14 (0.032–0.5995)	0.008 **

* significant; ** high significant; *** very high significant.

## Data Availability

Data is available upon request from the first and corresponding author.
